# Integrating Wearable Sensors and Video to Determine Microlocation-Specific Physiologic and Motion Biometrics-Method Development for Competitive Climbing

**DOI:** 10.3390/s22166271

**Published:** 2022-08-20

**Authors:** Miyuki Breen, Taylor Reed, Hannah M. Breen, Charles T. Osborne, Michael S. Breen

**Affiliations:** 1 Department of Mathematics, North Carolina State University, Raleigh, NC 27695, USA; 2 The Beta Angel Project, Alexandria, VA 22304, USA; 3 Sportrock Performance Institute, Alexandria, VA 22304, USA; 4 Department of Biomedical Engineering, The University of Utah, Salt Lake City, UT 84112, USA; 5 Department of Civil, Construction, and Environmental Engineering, North Carolina State University, Raleigh, NC 27695, USA

**Keywords:** physiologic monitoring, video analysis, heart rate, breathing rate, minute ventilation, physical activity, rock climbing, indoor climbing

## Abstract

Competitive indoor climbing has increased in popularity at the youth, collegiate, and Olympic levels. A critical aspect for improving performance is characterizing the physiologic response to different climbing strategies (e.g., work/rest patterns, pacing) and techniques (e.g., body position and movement) relative to location on climbing wall with spatially varying characteristics (e.g., wall inclinations, position of foot/hand holds). However, this response is not well understood due to the limited capabilities of climbing-specific measurement and assessment tools. In this study, we developed a novel method to examine time-resolved sensor-based measurements of multiple personal biometrics at different microlocations (finely spaced positions; MLs) along a climbing route. For the ML-specific biometric system (MLBS), we integrated continuous data from wearable biometric sensors and smartphone-based video during climbing, with a customized visualization and analysis system to determine three physiologic parameters (heart rate, breathing rate, ventilation rate) and one body movement parameter (hip acceleration), which are automatically time-matched to the corresponding video frame to determine ML-specific biometrics. Key features include: (1) biometric sensors that are seamlessly embedded in the fabric of an athletic compression shirt, and do not interfere with climbing performance, (2) climbing video, and (3) an interactive graphical user interface to rapidly visualize and analyze the time-matched biometrics and climbing video, determine timing sequence between the biometrics at key events, and calculate summary statistics. To demonstrate the capabilities of MLBS, we examined the relationship between changes in ML-specific climbing characteristics and changes in the physiologic parameters. Our study demonstrates the ability of MLBS to determine multiple time-resolved biometrics at different MLs, in support of developing and assessing different climbing strategies and training methods to help improve performance.

## 1. Introduction

Rock climbing is a fast-growing international sport with the number of indoor climbing gyms increasing each year [1,2]. As a result, the USA Climbing governing body was founded in 1998, the International Federation of Sport Climbing (IFSC) was formed in 2007, and competitive climbing debuted at the Tokyo 2020 Summer Olympics [2]. According to the IFSC, there are 44.5 million climbers worldwide [1].

The three events in youth and adult indoor climbing competitions, including the Olympics, are bouldering, lead climbing, and speed climbing [2,3]. In brief, bouldering refers to climbing a wall typically 4–5 m high without a rope, and climbers have multiple attempts to complete the climb in four min. Lead climbing refers to ascending a wall typically 15–20 m high with a rope that the climber attaches to fixed anchors along the route. The climber has one attempt to complete the route in 6–8 min. For bouldering and lead climbing, the position and type of hand and foot holds used to ascend the wall are different for each competition and unknown to the competitor, and therefore cannot be rehearsed or specifically trained for [3]. The climber is scored based on the amount of route completed. Finally, speed competitions take place on a 15 m standardized climbing wall, and identical holds and route are used each time for consistency across competitions [2].

The recent surge in interest in climbing has generated a need to maximize climbing performance through the design of specific physical and mental training and conditioning [4,5,6,7,8,9,10]. Knowledge of individual physiologic responses during a climb is useful to prescribe personalized exercises, provide instruction, and evaluate training methods [11,12,13,14]. The contribution of physiologic biometrics to performance can vary with the type and difficulty of the climb. The factors that can affect the difficulty of a climb include type and positioning of hand and foot holds (route settings), inclination of wall, and length of route [15,16,17,18,19].

A critical aspect for improving performance of competitive climbers is characterizing the physiologic response to different climbing strategies (e.g., work/rest patterns, pacing) and techniques (e.g., body position and movement) relative to location on the climbing wall with spatially varying characteristics (e.g., wall angles, boulder and lead route settings) [12,13,14,15,16]. However, this response is not well understood due to the limited capabilities of climbing-specific measurement and assessment tools [15,16,17,18,19,20]. In this study, we developed a novel method to examine time-resolved sensor-based measurements of multiple biometrics at different microlocations (finely spaced positions; MLs) along a climbing route, which is called the ML-specific biometric system (MLBS). The MLBS consists of (1) biometric sensors that are embedded in the fabric of an athletic compression shirt and do not interfere with climbing performance, (2) third-person video of the climber ascending a wall, and (3) an interactive graphical user interface (GUI) to rapidly visualize and analyze three physiologic parameters (heart rate (HR), breathing rate (BR), minute ventilation (V_E_)), one body movement parameter (hip acceleration (HA)), and the climbing video.

This manuscript demonstrates the capabilities of MLBS for application in future climbing panel studies. We first describe the data acquisition procedure, and then the visualization and analysis system to determine time-resolved ML-specific biometrics and to examine the relationship between changes in ML-specific climbing characteristics and changes in the physiologic parameters.

## 2. Materials and Methods

### 2.1. Respiratory and Cardiac Measurements (BR, V_E_, HR)

The two respiratory parameters (breathing rate, BR; minute ventilation, V_E_) and one cardiac parameter (heart rate, HR) are obtained from the Hexoskin biometric compression shirt (Carre Technologies Inc., Montreal, Quebec, Canada) every 1 s. The BR, V_E_, and HR for the biometric shirt were previously evaluated using laboratory standard devices, which included breath-by-breath flow sensors to measure BR and V_E_; and electrocardiogram (ECG) monitors to measure HR [21,22,23,24]. Elliot et al. showed mean absolute errors of 3% for BR and HR, and 5–8% for V_E_ during moderate-intensity exercise (50–75% maximum work rate during stationary cycling) [21]. Villar et al. showed mean absolute errors of 2% for BR and 1% for HR during moderate- and high-intensity exercise (80% ventilation threshold test, 80% predicted maximum HR) [22]. The shirt is constructed from a high-performance fabric (73% polyamide microfibers, 27% elastane), and contains respiratory and cardiac sensors sewn directly into the fabric in thoracic and abdominal bands. The shirts are available in different sizes for both men and women to ensure the proper fit based on the manufacturer’s suggested chest and waist circumferences. For the women’s shirts, the thoracic band is built into the lower band of the sports bra, whereas the abdominal band is built into the main shirt fabric. For the men’s shirts, both the thoracic and abdominal bands are both built into the main shirt fabric. The manufacturer-supplied elastic straps are adjusted and positioned to hold both the thoracic and abdominal sensors firmly against the skin to help reduce any possible motion artifacts due to displacement of the sensors, which could occur with upper body movements.

The biometric shirts continuously measure BR (breaths/min), tidal volume (L/inspiration, V_T_), and HR (beats/min), which are automatically time-matched and stored by a data logger that is secured in a sewn-in pouch in the compression shirt near the right hip. The data logger connects to the cardiac and respiratory sensors in the shirt via a cable in the pouch. The V_E_ (L/min), which is the volume of air inhaled per minute, is the product of V_T_ and BR. The BR and V_T_ are estimated via dual channel respiratory inductance plethysmography (RIP) sensors. The RIP sensors are strain gauges that collect inspiration and expiration displacements of the thoracic and abdominal bands at 128 Hz, which are used to derive V_T_ and BR every 1 s. The V_T_, BR, and body weight are used to estimate V_E_ every 1 s. The BR and V_E_ are averages across 5 and 7 respiratory cycles, respectively, which are fixed values set by the manufacturer. 

For HR measurements, the biometric shirts are embedded with three textile electrodes (two at the thoracic level, one at the abdominal level near the right hip) to yield one-lead ECG recordings at 256 Hz, which are used to derive HR every 1 s. The HR measurements are averaged across 16 heart beats, which is a fixed value set by the manufacturer.

### 2.2. Hip Acceleration (HA) Measurements

The body movement parameter (hip acceleration, HA) is obtained from the Hexoskin accelerometer every 1 s. The HA for this accelerometer was previously evaluated and shown to be reliable with a mean absolute error of 2% [22]. The accelerometer is embedded within the data logger, which automatically time-matches the HA data with the respiratory and cardiac data, as described above. The data logger has 36 h of battery life and 100 days of memory to allow for long recording sessions. The three-axis accelerometer continuously measures the acceleration in G-forces (g) in the x, y, and z directions at 64 Hz, which is a fixed sampling rate set by the manufacturer. The x, y, and z measurements are combined to determine the magnitude of acceleration (HA). The HA are averaged across 1 s, which is a fixed value set by the manufacturer.

### 2.3. Climbing Video Measurements and Time-Matching with Biometrics Data

A video of the climber is collected from the floor with a smartphone (iPhone11, Apple Inc., Cupertino, CA, USA). It should be noted that any digital video recording device can be used. The video frame rate is 30 frames/s. The climbing video is time-matched to the biometric data using a calibration protocol. The calibration is performed by starting the collection of video and biometric data while the climber is on the floor. The climber then ascends the wall approximately 30 cm before dropping to the floor. This drop produces a large transient spike in the HA data that is manually time-matched to the video frame at the corresponding time using MATLAB software (version R2019a; MathWorks, Natick, MA, USA). 

**Figure 1:**
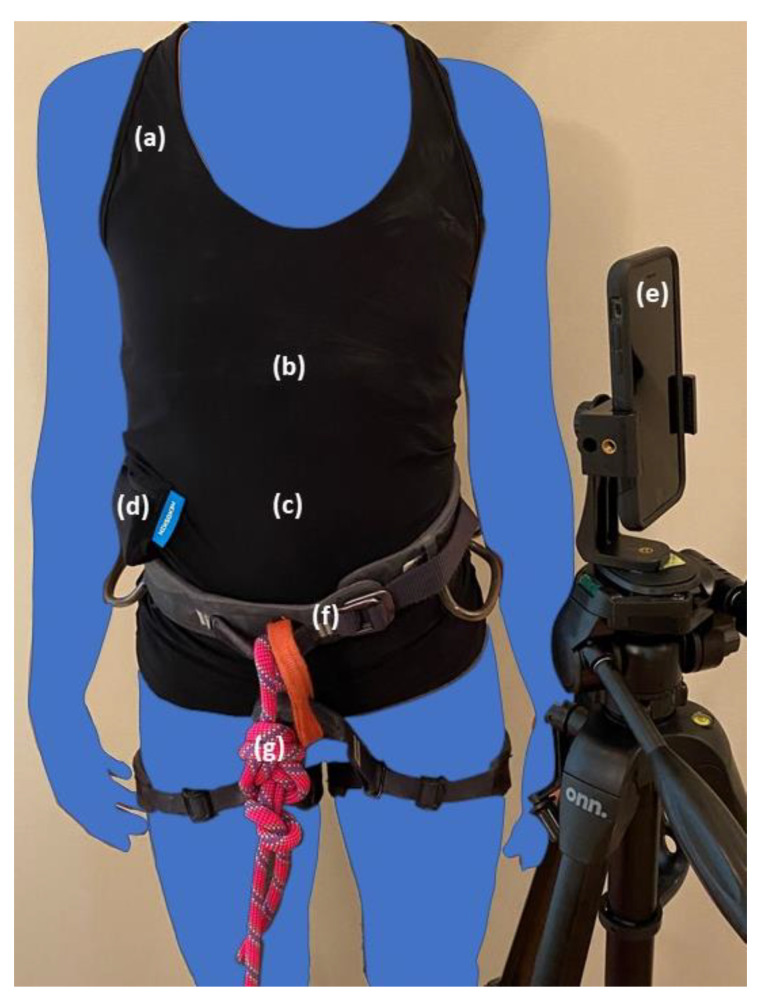
Setup for collecting biometrics and video of a lead climb, which includes: (**a**) biometric compression shirt; (**b**,**c**) thoracic and abdominal bands with respiratory and cardiac sensors; (**d**) compression shirt pouch with data logger and accelerometer; (**e**) smartphone for climbing video; (**f**) climbing harness; and (**g**) climbing rope.

To demonstrate the visualization and analysis features of MLBS, we used one demonstration input sample (lead climb) provided from one author who is a highly experienced climber, has competed in national-level championships for many years, and uses Hexoskin and climbing video data for training. The data were collected using the same method as described above. Figure 1 shows the setup to collect the four biometrics and climbing video for a lead climb.

### 2.4. Design and Operation of Visualization and Analysis System

**Figure 2:**
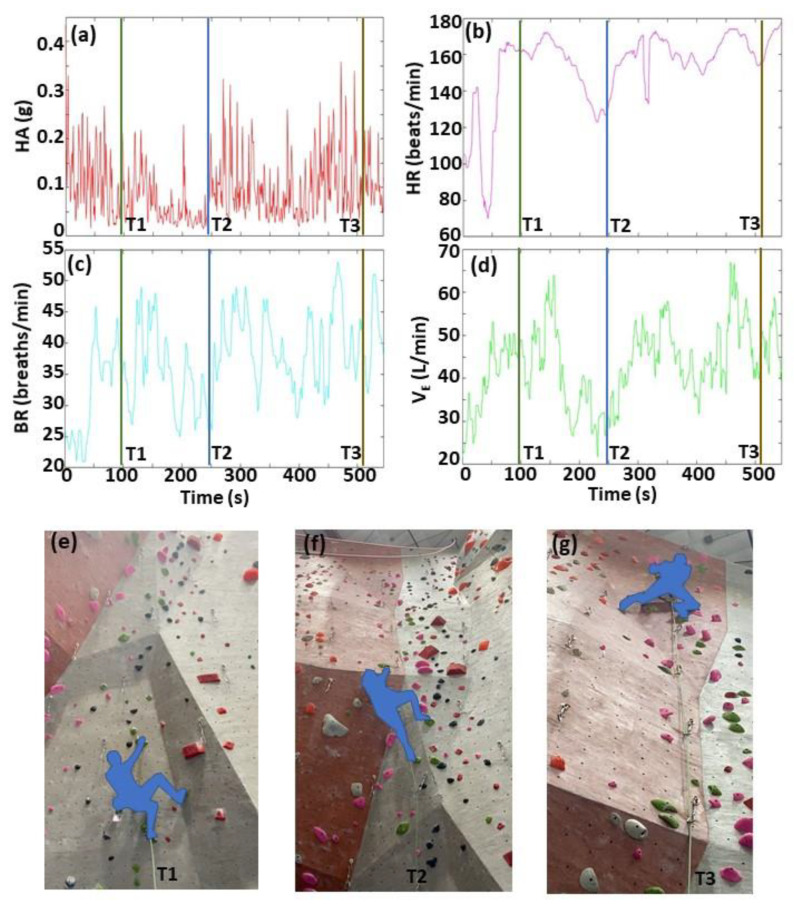
Demonstration of Menu Option 1—selection of time points in biometrics window, and display of corresponding climber video frames. The four biometrics include: (**a**) hip acceleration (HA); (**b**) heart rate (HR); (**c**) breathing rate (BR); (**d**) minute ventilation (V_E_). Climbing video frames (**e**–**g**) show three locations of a lead climber while ascending an indoor climbing wall, which correspond to the three time points (T1–T3) in the biometrics data, respectively. After the user positions a cursor in the biometrics window and clicks at a time point in one biometric plot, the software immediately displays the time-matched climbing video frame, and displays time-markers at the selected time point (vertical lines) in all four biometric plots.

**Figure 3:**
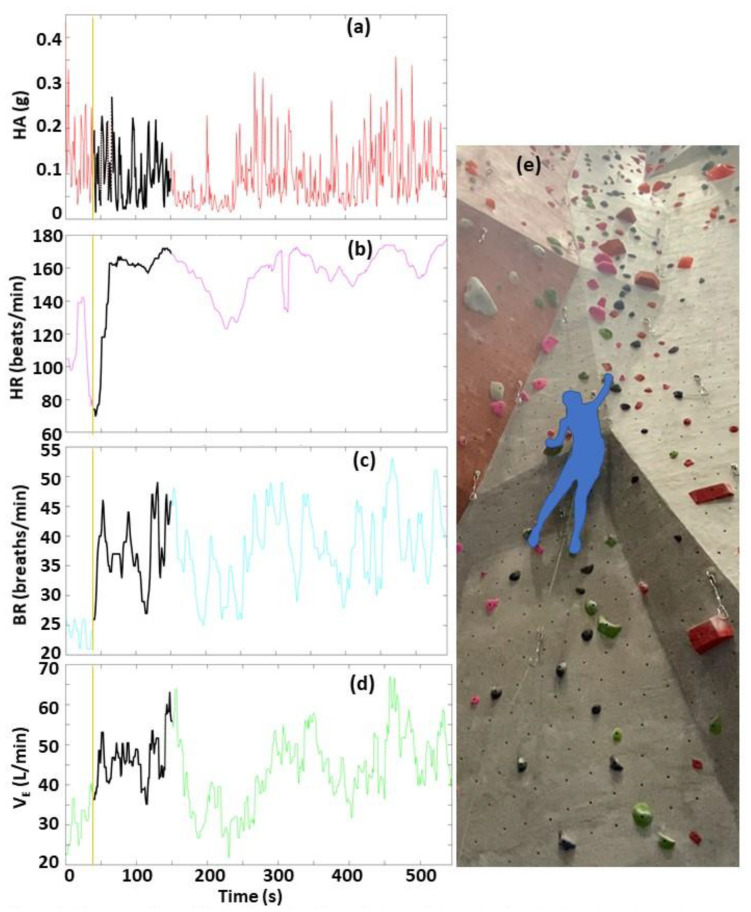
Demonstration of Menu Option 1—video playback of biometrics with a black line tracing the data across time (**a**–**d**), and time-matched climbing video (**e**). The rightmost leading edge of the black line corresponds to current climbing video frame (**e**). The orange vertical line corresponds to the starting time of the video playback. To select the starting time, the user positions a cursor in the biometrics window, and clicks at the desired time point (as shown in Figure 2). The software then starts the video playback.

**Figure 4:**
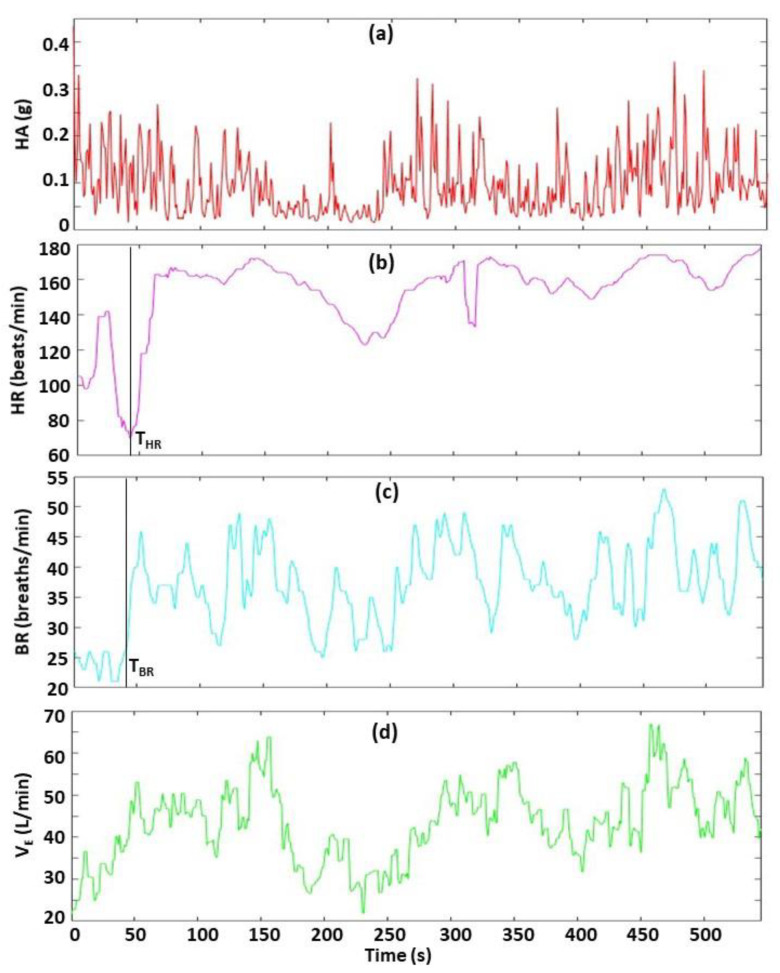
Demonstration of Menu Option 2—determine timing sequence of events from biometrics. The four biometrics are HA (**a**), HR (**b**), BR (**c**) and V_E_ (**d**). In this example, the user selected time points T_HR_ and T_BR_ to specify the times immediately before a rapid increase in HR and BR, respectively, as shown by the black vertical lines. The user positions a cursor in the window displaying HR data and clicks at T_HR_, and then places the cursor in the window displaying the BR data and clicks at T_BR_. Then, the software automatically determines and displays the results, which show T_BR_ occurs 2.9 s before T_HR_.

**Figure 5:**
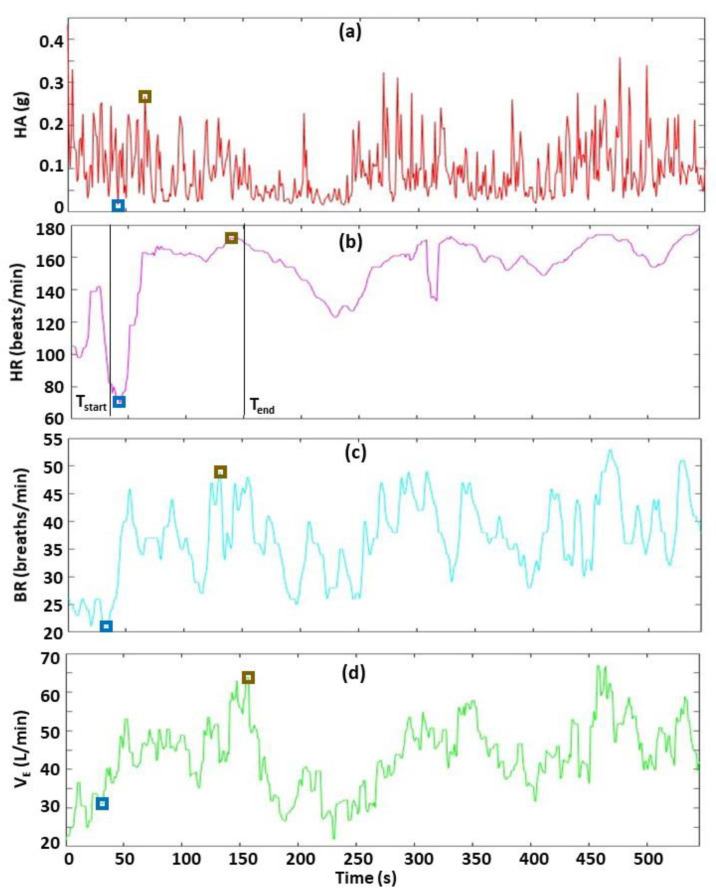
Demonstration of Menu Option 3—calculate summary statistics for biometrics. The four biometrics are HA (**a**), HR (**b**), BR (**c**) and V_E_ (**d**). In this example, the user selected a time range (T_start_—T_end_) that has substantial changes in HR. The user positions a cursor in the window displaying the HR data, and clicks at two time points (T_start_, T_end_) to define the time range (black vertical lines). Then, the software automatically calculates and displays the statistics for all four biometrics (HR, BR, V_E_, HA) in this time range. The blue and brown boxes indicate the time when minimum and maximum values occur in this time range, respectively.

To facilitate the visualization and analysis of the five large time-matched data sets (four biometrics, one video), we developed an interactive GUI using MATLAB software. The GUI displays two windows: one for the biometrics, and another for the time-matched climbing video, as shown in Figure 2. The user selects from three menu options: (1) selection of time point in biometrics window to display of corresponding climbing video frame (Figure 2), and to play video of biometrics with moving time-marker synchronized with climbing video (Figure 3); (2) determination of timing sequence of events from biometrics (Figure 4); and (3) calculation of summary statistics of biometrics (Figure 5). 

Menu Option 1 (Figure 2 and Figure 3) allows the user to easily interact with the climbing video and biometrics data. A key feature is the ability to rapidly locate and analyze a region of interest (ROI) based on either a specific location in the climbing video (e.g., start of a large inclination in the wall) or a specific event in the biometrics data (e.g., sudden increase in HR). To locate this ROI, the user places a cursor in the biometrics window and clicks a time point in one of the four biometrics plots. The software then immediately (1) updates and displays the time-matched climbing video frame, and (2) displays a corresponding time-marker in all four biometric plots (Figure 2). The user can repeat this process until the desired video frame is displayed, and then the software displays a video that starts at this user-defined video frame. Two synchronized (time-matched) videos are displayed simultaneously (Figure 3). One is the climbing video. The other is a video of the biometric time-course data with a black line tracing the data across time, as shown in Figure 3. The playback video frame rate is set to slow motion (6 frames/s, 20% of normal playback speed) to allow for a more careful video analysis. 

For Menu Option 2 (Figure 4), the user selects time points in the biometrics window to examine the chronological order for events of interest (EOIs). For example, this feature can be used to determine the time difference between a sudden increase in HR, BR, and V_E_. The user positions a cursor in the biometrics window to select an EOI in two or more biometric plots, and the software determines and displays the chronological order and the time difference between the EOI. 

For Menu Option 3 (Figure 5), the user positions a cursor in the biometrics window to specify the time range for calculating summary statistics. The summary statistics include minimum, median, maximum, and time when minimum and maximum occur.

## 3. Results 

**Figure 6:**
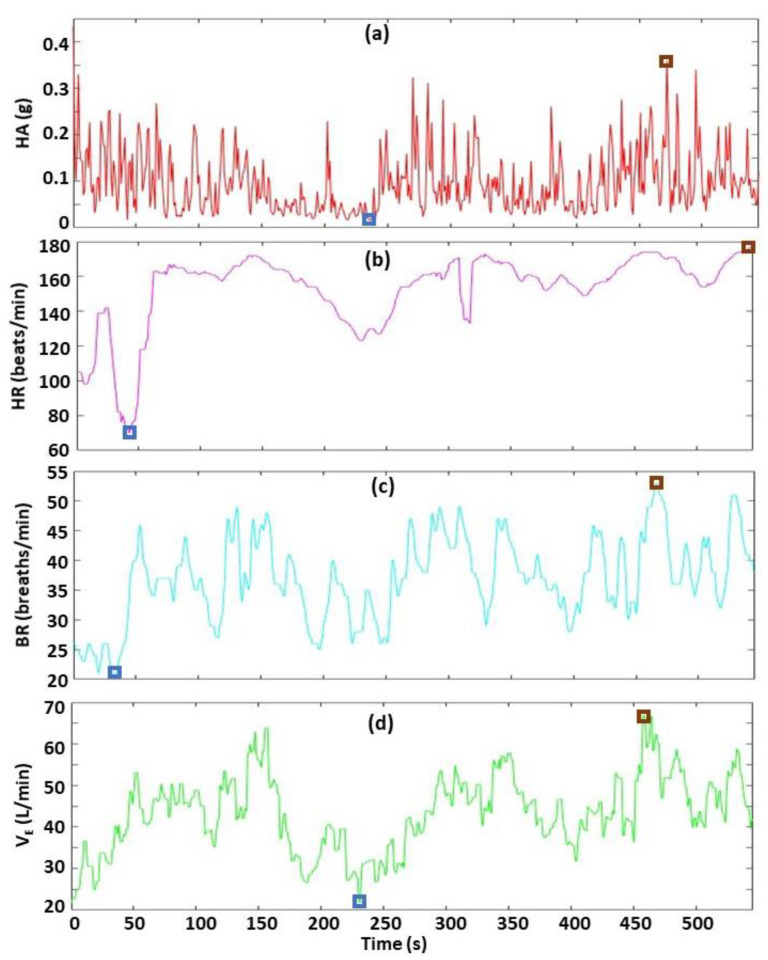
Characterization of the biometrics across entire climbing time. The four biometrics are HA (**a**), HR (**b**), BR (**c**) and V_E_ (**d**). The blue and brown boxes indicate the minimum and maximum values, respectively. The large spike in HA at time = 0 is the calibration sample used to time-match the biometric data with the climbing video (details provided in Methods Section 2.3). This HA data point is excluded from the summary statistics (e.g., minimum, maximum) since it occurs when the climber intentionally drops 30 cm to the floor, immediately before starting the climb.

Figure 6 shows the four biometrics (HA, HR, BR, V_E_) during a 530 s (8:50, min:sec) lead climb on a 16.8 m (55 ft) indoor climbing wall. The HA show large transient spikes (max HA = 0.36 g) with short periods with sustained lower HA (0.01–0.08 g). Using the GUI with the time-matched climbing video, we found that the large spikes occurred when actively climbing (due to hip movements while ascending the route), and the sustained lower HA correspond to resting. Moreover, larger body movements while climbing corresponded to higher HA. The HA could be further examined to determine work:rest ratios. In a different demonstration sample (Supplementary Material, Figure S1), the HA = 1.0 g during a lead climb fall, which is a good indicator that the HA values are reasonable. 

The HR showed substantial variability with a range between 70 and 174 beats/min (min–max), and a median of 160 beats/min (Figure 6). This HR range compares closely to a previous lead climbing study that used wearable HR monitors [18]. Using MLBS, we found that the HR consistently showed increases when actively climbing, and decreases when resting on the wall. We also found that HR increases when climbing difficulty increases (based on opinion of the climber), which may be due to increases in physical energy needs or psychological factors. This demonstrates that MLBS can be applied in future panel studies to help determine the factors that influence HR changes (e.g., physical energy needs, psychological factors) in support of helping coaches develop personalized training plans.

The BR varied between 21 and 53 breaths/min, with a median of 37 breaths/min (Figure 6). Using MLBS, we found that BR increases often when climbing but also sometimes when resting on the wall. Moreover, the BR sometimes decreases when climbing. This indicates that BR changes may be affected by physical energy needs, psychological factors, and tactical choices (e.g., pacing choices such as slowing movement). This can be examined in future panel studies. 

We also visually compared changes in BR and HR. The BR and HR increases (and decreases) tend to occur simultaneously. However, sometimes the BR decreases and the HR increases when actively climbing, which can be explored in a future study. 

The V_E_ varied between 22 and 67 L/min, with a median of 44 L/min (Figure 6). Overall, V_E_ and BR show similar temporal trends with corresponding increases and decreases for both biometrics across several time periods. Moreover, the minimum V_E_ and BR occurred at approximately the same time (457, 466 s), respectively. Since V_E_ is the product of BR (breaths/min) and V_T_ (L/breath), V_E_ changes relative to BR changes can be an indicator of changes in V_T_ (i.e., deep/shallow breathing). 

To demonstrate the capabilities of MLBS, we analyzed the biometrics during a climbing period with substantial variations in HR, BR, and V_E_ that occurred between 46 and 151 s (Figure 5). During this time period, the climber had difficulty ascending this section of the climbing route, and needed to try several different climbing strategies (e.g., variations in hold sequences, body movements) before completing this section without a fall. This period had remarkably large short-term increases in HR (70–172 beats/min; 146% increase), BR (26–49 breaths/min; 88% increase), and V_E_ (25–63 L/min; 152% increase), as compared to other sections of the climbing route. Following this difficult climbing section, the climber rested and the HR decreased to 123 beats/min, which is only a 50% return to its initial level of 70 beats/min, but the BR and V_E_ decreased completely to their initial levels.

## 4. Discussion

Our goal was to develop and demonstrate a method to determine four time-resolved sensor-based biometrics (HA, HR, BR, V_E_) from a non-invasive athletic compression shirt integrated with time-matched climbing video from a smartphone. The visualization and analysis system was used to compare changes in the physiologic response with different climbing strategies (e.g., work/rest patterns, pacing) and techniques (e.g., body position, movement) relative to location on the climbing wall with spatially varying characteristics (e.g., wall angles, hold settings). Our results demonstrate the feasibility of using time-resolved non-invasive sensor data with climbing video to determine ML-specific biometrics, in support of developing and evaluating strategies to improve climbing performance.

We can compare our results of measured HR and V_E_ with other climbing studies. Previous studies show that HR increases as climbing difficulty increases, and reported maximum HR during lead climbing between 129 and 180 beats/min [4,7,8], which compares closely to our study with a maximum HR of 174 beats/min. The large increases in HR during climbing are due to: (1) repetitive isometric forearm contractions that activate muscle metaboreflex and induce HR increases, (2) climbing with arms held above the level of the heart, which is associated with HR increases, and (3) psychological factors (e.g., fear of falling) [4,7,8]. For V_E_, previous studies show that V_E_ (i.e., whole body oxygen consumption) increases during climbing [4,5,7], and reported V_E_ of 37 ± 11 L/min (mean +/− SD) [7], which compares reasonably to our study with a median V_E_ of 44 L/min. Increases in V_E_ indicate that lead climbing requires a significant portion of whole body aerobic (with oxygen) capacity, together with anaerobic (without oxygen) energy pathways [4,5,7,8,12].

To demonstrate the capabilities of MLBS, we used the Hexoskin wearable biometric device. To the best of our knowledge, Hexoskin is currently the only commercially available device that can measure all four biometrics (HR, BR, V_E_, HA) with sensors that are seamlessly embedded in a non-invasive comfortable athletic compression shirt. Furthermore, this biometric device has been evaluated and applied in several previous studies [21,22,23,24]. It is also important to note that our overall method, including our visualization and analysis system, can be applied for any type of wearable biometric sensor system in future studies. 

There are other wearable biometric sensor systems that may be integrated with the MLBS in future studies. First, previous climbing studies used mobile breath-by-breath spiroergometry devices (e.g., MetaMax 3B, Cortex Biophysic, Germany) to directly measure V_E_, oxygen intake, and carbon dioxide output [5,7,9]. The ability to measure oxygen and carbon dioxide expands the capability of analyzing additional physiological responses while climbing. However, unlike our study’s non-invasive Hexoskin biometric shirt with textile sensors embedded into a comfortable garment, wearing a spiroergometry device with a completely air-sealed face mask and a chest/back carrying unit likely has a negative impact on climbing performance. This impact is due to various factors, including the weight of the device, potential discomfort and interference with natural body movement, and limited ability to explore the wall while climbing due to the breathing apparatus protruding from the mouth/nose. Second, MLBS can be integrated with video data from mobile eye tracking glasses to determine what the climber is looking at as they ascend the wall [25,26]. Eye tracker data may help improve climbing skills by examining the amount of time spent exploring different climbing options and hold sequences, and the time spent looking at placement of feet and hands on the holds. Finally, MLBS may be integrated with mobile galvanic skin response sensors to measure changes in sweat gland activity that can indicate the intensity of a climber’s emotional arousal and other psychological responses during climbing [27].

The MLBS has several potential applications. First, since the Hexoskin biometric garment is a completely non-invasive and comfortable athletic compression shirt, MLBS can be applied for bouldering, lead climbing, and speed climbing without impacting climbing performance. Second, HR and BR patterns could be examined for different climbing situations and while resting. MLBS could examine whether climbers are breathing sufficiently, and investigate if induced breathing during difficult climbing conditions (e.g., overhanging wall, locations with potentially large falls) or during speed climbing can enhance performance. Knowledge of respiration and movement can help improve climbing performance [20]. Third, MLBS could be used to examine training interventions associated with resting (i.e., increasing/decreasing length or location of rests) or interventions associated with pacing (i.e., increasing/decreasing climbing speed) based on wall angle, movement difficulty, movement type, or route hold affordances (i.e., size, angle, inclination, relationship with other holds). Finally, MLBS could help determine the amount and rate of recovery for HR, BR, and V_E_ while resting between climbs.

In future studies, MLBS can be applied for panel studies with climbers of different genders and skill levels. The studies can examine both indoor climbing (bouldering, lead climbing, speed climbing) and outdoor climbing (bouldering, lead climbing). The MLBS may also be used as a training tool for belayers. When a lead climber falls, a climbing partner called a belayer manually applies tension at the other end of the climber’s rope to minimize the distance a climber falls while slowly decreasing the acceleration of the climber as they fall (i.e., providing a soft catch). A soft catch distributes the forces impacting the climber to avoid injuries. The MLBS can be used to examine the effectiveness of using HA and the climbing video during lead climbing falls to characterize a soft catch and thereby help to improve the performance of belayers.

There are some limitations to our study. One limitation is that the Hexoskin device cannot directly measure V_E_ but instead provides an estimate of V_E_ based on BR, V_T_, and body weight. For future studies, we plan to use a pneumotach, which is a laboratory instrument that directly measures inhaled volumes, to perform a calibration of V_E_ for each climber. Another limitation is that the Hexoskin device cannot wirelessly transmit the respiratory biometrics (BR, V_E_) while climbing. Therefore, for real-time applications, alternative wearable devices can be used with real-time wireless communication capabilities, such as the BioNomadix respiration transducer and wireless transmitter (BioPac Systems, Goleta, CA, USA).

## 5. Conclusions

Our study demonstrates the ability to determine time-resolved sensor-based measurements of multiple biometrics (HA, HR, BR, V_E_) at different locations along a climbing route, in support of developing and assessing different climbing strategies and training methods to help improve performance. The two key features of MLBS are (1) the non-invasive respiratory, cardiac, and movement sensors that are seamlessly integrated in a high-quality athletic compression shirt that does not interfere with climbing performance, and (2) the interactive GUI to rapidly visualize and analyze a time-matched climbing video with the biometric sensor data. Using MLBS, we demonstrated the ability to examine relationships between changes in location-specific climbing characteristics (e.g., difficult climbing sections on the wall) and corresponding changes in biometrics.

## Data Availability

Data is available from corresponding author.

## Supplementary Materials

The following supporting information can be downloaded at: https://www.mdpi.com/article/10.3390/s22166271/s1, Figure S1. Hip acceleration during a lead climb with a fall.
