# Supplementary material for: Integrating Wearable Sensors and Video to Determine Microlocation-Specific Physiologic and Motion Biometrics-Method Development for Competitive Climbing
Source: Sensors (Basel). 2022 Aug 20;22(16):6271. doi: 10.3390/s22166271 (PMC9412409; doi:10.3390/s22166271)
Supplement: Supplementary file 1 [file sensors-22-06271-s001.zip › sensors-1847262-supplementary.pdf]

sensors

Article

Integrating Wearable Sensors and Video to Determine Microlocation-Specific Physiologic and Motion Biometrics-Method Development for Competitive Climbing

Miyuki Breen [1,*], Taylor Reed [2,3], Hannah M. Breen [2], Charles T. Osborne [2,4] and Michael S. Breen [5]

[1] Department of Mathematics, North Carolina State University, Raleigh, NC 27695, USA
[2] The Beta Angel Project, Alexandria, VA 22304, USA
[3] Sportrock Performance Institute, Alexandria, VA 22304, USA
[4] Department of Biomedical Engineering, The University of Utah, Salt Lake City, UT 84112, USA
[5] Department of Civil, Construction, and Environmental Engineering, North Carolina State University, Raleigh, NC 27695, USA
* Correspondence: mbreen@ncsu.edu

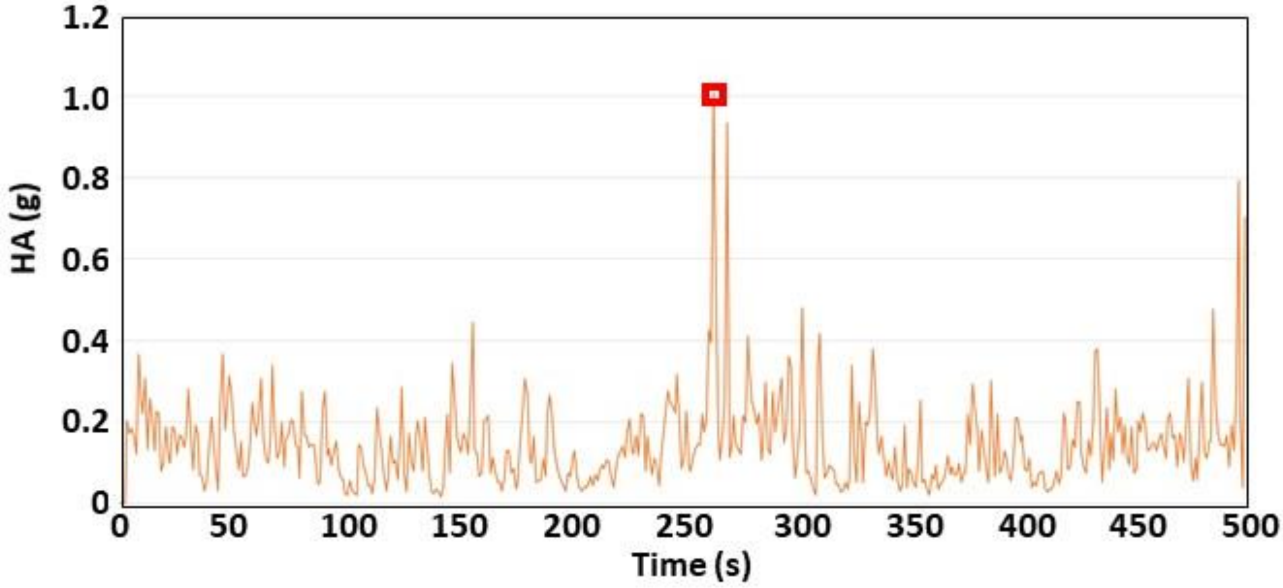

Figure S1. Hip acceleration (HA) during a lead climb with a fall. The red square indicates the time when the lead fall occurs and HA = 1.0 g.
